# Associations between anthropometric parameters and lipid profiles in Chinese individuals with age ≥40 years and BMI <28kg/m^2^

**DOI:** 10.1371/journal.pone.0178343

**Published:** 2017-06-20

**Authors:** Zhi Yang, Xun Ding, Jiang Liu, Peng Duan, Lian Si, Binghua Wan, Ping Tu

**Affiliations:** 1 Nanchang Key Laboratory of Diabetes, The Third Hospital of Nanchang, Jiangxi Province, China; 2 Department of Endocrinology and Metabolism, The Third Hospital of Nanchang, Jiangxi Province, China; Beijing Key Laboratory of Diabetes Prevention and Research, CHINA

## Abstract

**Background:**

Lipid abnormalities are associated with overweight and obesity. Some simple anthropometric measurements such as body mass index (BMI), waist circumference (WC), waist-to-hip ratio (WHpR), and waist-to-height ratio (WHtR), may link to increased risk of dyslipidemia. However, diverse results were found in different population studies. We focused on the associations between these measurements and dyslipidemia in non-obese (BMI <28kg/m^2^) population aged more than 40 years.

**Methods and findings:**

Cross-sectional study of 4185 non-obese adults aged more than 40 years was conducted in Nanchang, Jiangxi province, China. Questionnaire, anthropometric and laboratory tests were conducted. The National Cholesterol Education Program (NCEP) Expert Panel on Detection, Evaluation, and Treatment of High Blood Cholesterol in Adults (Adult Treatment Panel III) criteria were used to define high total cholesterol (TC), high low-density lipoprotein cholesterol (LDL-C), low high-density lipoprotein cholesterol (HDL-C), hypertriglyceridemia and dyslipidemia. The overall prevalence of high TC, high LDL-C, low HDL-C, hypercholesterolemia, hypertriglyceridemia and dyslipidemia were 15.68%, 27.98%, 20.12%, 44.01%, 21.98% and 49.06% respectively. Multiple logistic regressions showed only BMI (per quartile increment) increased risks for prevalent high LDL-C, low HDL-C, hypercholesterolemia, hypertriglyceridemia, and dyslipidemia. Regardless of sex, age and prevalent metabolic syndrome, increasing BMI was persistently independent risk factor for having low HDL-C, hypercholesterolemia and dyslipidemia, however was not associated with high TC.

**Conclusions:**

In non-obese Chinese population aged more than 40 years, increasing BMI may better identify the prevalent dyslipidemia than other anthropometric measurements. However, due to the different meanings, both BMI and WC should be measured and monitored for metabolic risk assessment.

## Introduction

Prevention of coronary heart disease (CHD) is becoming an increasingly serious public health issue worldwide. Dyslipidemia is considered as one of the most important modifiable risk factors for CHD [1–3]. Therefore, early diagnosis and active treatment of dyslipidemia are excellent precautions for CHD. Dyslipidemia mainly refers to hypercholesterolemia and/or hypertriglyceridemia, the former contains high total cholesterol (TC) and/or high low-density lipoprotein cholesterol (LDL-C) and/or low high-density lipoprotein cholesterol (HDL-C). In current Chinese adult population, almost 88.1 million persons had high TC, 62.8 million persons had high LDL-C and 214.9 million persons had low HDL-C in addition. The unsatisfactory awareness, treatment, and control call on effective intervention for dyslipidemia urgently, or else atherosclerotic cardiovascular diseases may soar in the near future in China [3].

In Adult Treatment Panel Ⅲ (ATP Ⅲ) from the National Cholesterol Education Program (NCEP), overweight and obesity are recognized as major, underlying risk factors for CHD and identified as direct targets of intervention. Elevated LDL-C, TG and reduced HDL-C levels are all associated with overweight and obesity [4]. Usually, some simple anthropometric measurements are used to reflect excess body fat, such as body mass index (BMI), waist circumference (WC), waist-to-hip ratio (WHpR), and waist-to-height ratio (WHtR). BMI is mostly used to define obesity. However, abdominal obesity evaluated by WC or WHtR has got more public attention recently. WC or WHtR are considered to be more correlated with metabolic risk factors than elevated BMI in several studies [5–7]. However, a study among 1278 children did not find particular superiority for WC, especially for WHtR based on routine measurement of BMI in predicting metabolic or cardiovascular risk [8]. It is still worth exploring the predictive effects by these anthropometric parameters in different population. In the present study, we compared the associations between various obesity indices and lipid profiles in a non-obese (BMI <28kg/m^2^) [9] Chinese population.

## Methods

The present cross-sectional study enrolled the subjects from communities and measured the obesity indexes and lipid concentrations.

### Ethics statement

The study procedure was approved by the Medical Ethics Committee of the Third Hospital of Nanchang. The written informed consent was obtained from each participant.

### Study population

**Fig 1:**
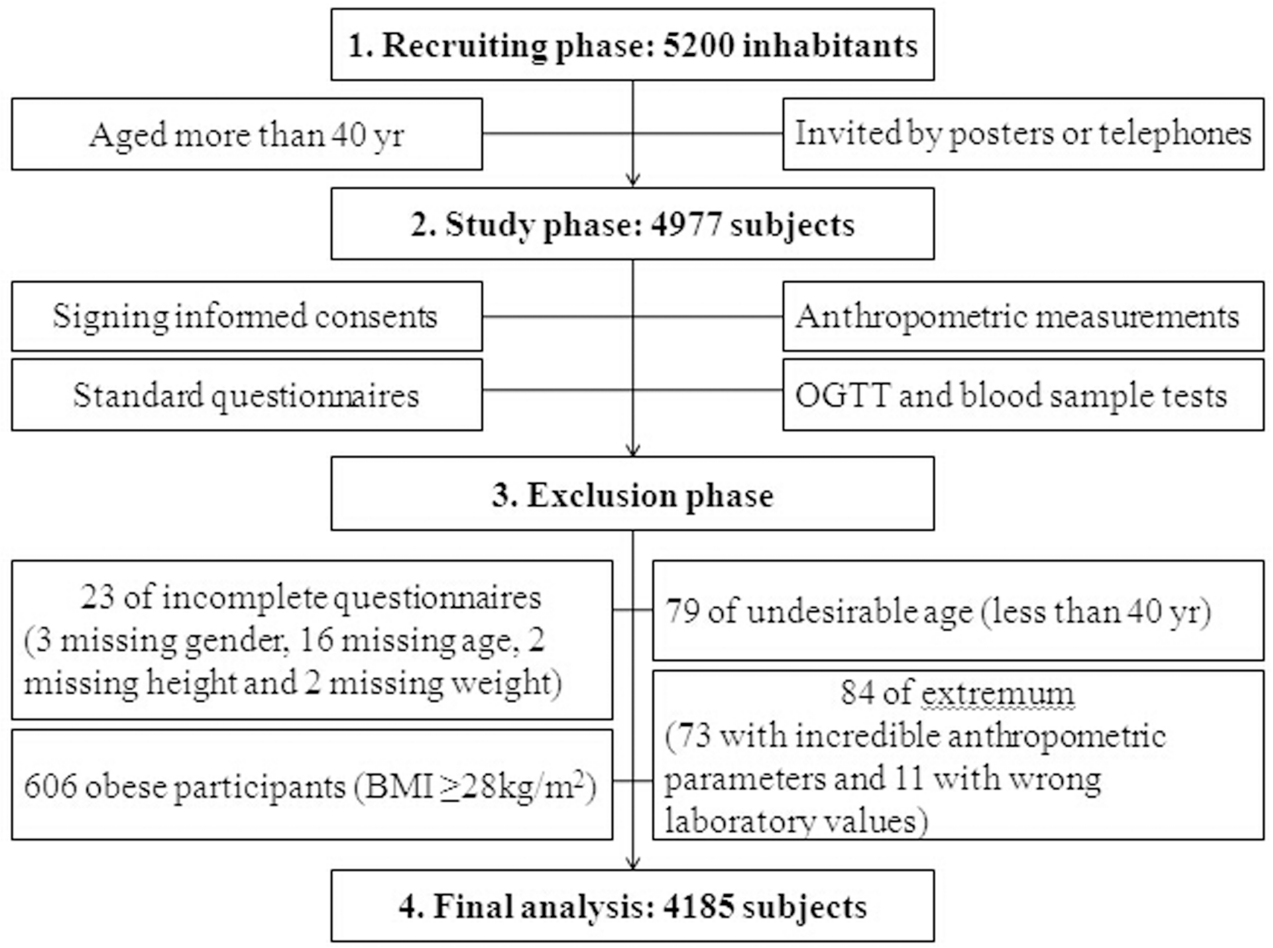
The flow diagram of study.

During March to July 2011, the study was conducted in Ximazhuang and Guangrunmen communities from Xihu district of Nanchang, Jiangxi province, China. In the recruiting phase, 5200 inhabitants aged more than 40 yr were invited by posters or telephones to participate in this program. We tried our best to obtain sufficient informed consent from inhabitants and avoid the age or gender bias as far as possible. From them, 4977 men and women attended to the study. Each subject had been registered with the identity card information. The deleted individuals included incomplete questionnaire (3 of them missing gender, 16 missing age, 2 missing height and 2 missing weight), undesirable age (79 of them aged less than 40 yr) and extremum (73 with incredible anthropometric parameters and 11 with wrong laboratory values). 606 obese participants (BMI ≥28kg/m^2^) were excluded from this study and at last, 4185 subjects were included in the final analysis (Fig 1).

### Data collection

A standard questionnaire was administered by trained staff to obtain information on demographic characteristics, medical history and lifestyle risk factors. Weight and height were determined in subjects wearing light clothing and no shoes. BMI was calculated as body weight in kilograms divided by body height squared in meters (kg/m^2^). WC was measured at the umbilical level in a standing position, whereas hip circumference (HC) was measured at the level of maximum extension of the buttocks. Twice sitting blood pressure measurements taken consecutively with 1-minute intervals using an automated electronic device (Omron Company, Dalian, China) were averaged for analysis.

### Laboratory measurements

All participants were under-taken a 75-g oral glucose tolerance test (OGTT) performed by a nurse. Blood samples were collected at 0 and 2 hours respectively by 2 specialized nurses. Serum TC, LDL-C, HDL-C and TG were measured using chemiluminescence methods, fasting blood glucose (FBG) and postprandial blood glucose (PBG) were measured using the glucose oxidase method on the same autoanalyser (Roche, Basel, Switzerland). The HbA1c level was measured by high-performance liquid chromatography (BIO-RAD Company, USA).

### Definition of adverse lipid concentrations and metabolic syndrome

Based on NCEP ATP Ⅲ criteria [4], TC ≥6.22 mmol/L was defined as high TC, LDL-C ≥4.14 mmol/L was defined as high LDL-C, HDL-C <1.04 mmol/L was defined as low HDL-C. Hypercholesterolemia contains high TC and/or high LDL-C and/or low HDL-C. TG ≥2.26 mmol/L was defined as hypertriglyceridemia. Dyslipidemia was defined as hypercholesterolemia and/or hypertriglyceridemia and/or use of lipid lowering medications.

Metabolic syndrome (MS) was diagnosed as the presence of three or more of the following abnormal factors: 1. TG ≥1.7 mmol/L; 2. HDL-C <1.0 mmol/L in men or <1.3 mmol/L in women; 3. Blood pressure ≥130/85 mmHg; 4. FBG ≥5.6 mmol/L or use of antidiabetic medications; 5. WC ≥85 cm in men or ≥80 cm in women [10].

### Statistical analyses

Cluster sampling method was used for the investigation. SAS 9.1 (SAS Institute, Cary, NC, USA) was used for all statistical analyses. Continuous variables were presented as means ± standard deviations (SD) or medians (interquartile ranges). FBG, PBG and TG levels were logarithmically transformed to achieve a normal distribution. All categorical variables were presented as numbers (proportions). The subjects were divided to different groups by sex. Furthermore, we explored some subgroups by stratifying individuals according to sex, age (<60 yr and ≥60 yr) and prevalence of MS. Comparisons of means and proportions were performed with Student’s *t*-test and Chi-squared test in two groups of men and women. Linear regression analysis and stepwise regression analysis were used to identify the linear correlations between anthropometric parameters and lipid profiles. The confounder-adjusted odds ratios (ORs) and 95% confidence intervals (CIs) were examined by logistic regression analysis. A P-value of less than 0.05 was considered to be statistically significant.

## Results

### Characteristics of the study population

The flow diagram is showed in Fig 1. The category boundaries of continuous variables are showed in Table 1. The present data included 4185 participants (33.52% men and mean age 61 ± 10 years). The overall prevalence of high TC, high LDL-C, low HDL-C, hypercholesterolemia, hypertriglyceridemia and dyslipidemia were 15.68%, 27.98%, 20.12%, 44.01%, 21.98% and 49.06% respectively. 423 subjects (10.11%) had lipid-lowering therapy. Compared with women, men had a significantly higher BMI, WC, WHpR, SBP, DBP, FBG, PBG, HbA1c and TG concentration, but smaller WHtR, lower TC, LDL-C and HDL-C concentrations. Besides, a higher proportion of men had the habit of smoking and had dyslipidemia, diabetes, hypertension and history of cardiovascular disease than women (all P-value <0.05). However, the comparison for having MS was not statistically different between both genders (P-value = 0.45) (Table 1).

**Table 1:** Characteristics of the study population.

	Category boundaries	Male	Female	P*-*value
**N (%)**	-	1403 (33.52)	2782 (66.48)	-
**Age (yr)**	40–95	63 ± 10	60 ±10	< .0001
**Smoker (n, %)**	-	486 (34.69)	61 (2.20)	< .0001
**BMI (kg/m**^**2**^**)**	13.10–27.99	23.57 ± 2.54	23.44 ± 2.53	< .0001
**WC (cm)**	58.00–113.00	84.75 ± 7.75	80.50 ± 8.20	< .0001
**WHpR**	0.67–1.33	0.91 ± 0.05	0.87 ± 0.06	< .0001
**WHtR**	0.34–0.73	0.51 ± 0.05	0.52 ± 0.05	< .0001
**SBP (mmHg)**	80–211	131 ± 19	128 ± 20	< .0001
**DBP (mmHg)**	38.5–127.5	77 ± 11	75 ± 10	< .0001
**FBG (mmol/L)**	2.49–22.25	4.79 (4.35–5.47)	4.75 (4.32–5.26)	< .0001
**PBG(mmol/L)**	2.30–32.35	6.21 (4.99–8.20)	6.01 (4.95–7.50)	0.0003
**HbA1c (%)**	3.5–15.5	6.0 ± 1.2	5.8 ± 0.8	< .0001
**TC (mmol/L)**	0.38–14.07	4.51 ± 1.08	4.88 ± 1.10	< .0001
**LDL-C (mmol/L)**	0.71–9.76	3.35 ± 0.91	3.50 ± 0.97	< .0001
**HDL-C (mmol/L)**	0.4–2.96	1.20 ± 0.32	1.39 ± 0.33	< .0001
**TG (mmol/L)**	0.30–23.13	1.24 (0.89–1.86)	1.19 (0.84–1.74)	0.0012
**Dyslipidemia (n, %)**	-	801 (57.09)	1252 (45.00)	< .0001
**Diabetes (n, %)**	-	295 (21.03)	400 (14.38)	< .0001
**Hypertension (n, %)**	-	663 (47.26)	1090 (39.19)	< .0001
**History of cardiovascular disease (n, %)**	-	62 (4.42)	88 (3.16)	0.039
**Having lipid-lowering therapy (n, %)**	-	143 (10.19)	280 (10.06)	0.91
**MS (n, %)**	-	416 (29.65)	856 (30.78)	0.45

Normal data were given as the mean ± SD, skewed data were given as medians (interquartile ranges) and categorical variables were given as numbers (proportions).

Abbreviations: BMI, body mass index; WC, waist circumference; WHpR, waist-to-hip ratio; WHtR, waist-to-height ratio; SBP, systolic blood pressure; DBP, diastolic blood pressure; FBG, fasting plasma glucose; PBG, 2h post-loading plasma glucose; TC, total cholesterol; LDL-C, low-density lipoprotein cholesterol; HDL-C, high-density lipoprotein cholesterol; TG, triglycerides; MS, metabolic syndrome.

### Linear correlations between anthropometric parameters and lipid profiles

Linear regression analysis revealed that sex, smoking status, BMI, WHtR, DBP and PBG were significantly related to TC, LDL-C, HDL-C and TG concentration. WC, WHpR, SBP and HbA1c were significantly related with LDL-C, HDL-C and TG concentration. Age was only significantly related with HDL-C and FBG was significantly related with TC, LDL-C and TG concentration (Table 2). Multiple stepwise regression analysis revealed that apart from traditional risk factors of adverse lipid concentrations, BMI, WC, WHpR and WHtR remained independent relationships with HDL-C. Besides, BMI was also an independent determinant of LDL-C and TG, WC and WHtR were also independent determinants of TC, WHpR was independent related to TG (Table 2).

**Table 2:** Linear correlations between anthropometric parameters and lipid profiles.

	TC				LDL-C				HDL-C				Log TG			
	*r*	P*-*value	*β*	P*-*value	*r*	P*-*value	*β*	P*-*value	*r*	P*-*value	*β*	P*-*value	*r*	P*-*value	*β*	P*-*value
**Age**	0.002	0.25			0.0009	0.53	0.0032	0.03	-0.0019	0.0003			-0.0019	0.36	-0.0014	0.01
**Sex (male = 1, female = 2)**	0.38	< .0001	0.25	< .0001	0.15	< .0001	0.14	< .0001	0.19	< .0001	0.11	< .0001	-0.11	0.02		
**Smoking (no = 0, yes = 1)**	-0.24	< .0001			-0.13	0.003			-0.15	< .0001			0.15	0.02		
**BMI**	0.022	0.001			0.042	< .0001	0.030	< .0001	-0.034	< .0001	-0.046	< .0001	0.068	< .0001	0.016	< .0001
**WC**	0.004	0.05	-0.022	0.001	0.0018	< .0001			-0.009	< .0001	-0.0078	0.0002	0.021	< .0001		
**WHpR**	0.18	0.52			0.60	0.01			-0.94	< .0001	-0.50	0.005	2.31	< .0001	0.28	0.002
**WHtR**	2.15	< .0001	4.55	< .0001	2.21	< .0001			-0.70	< .0001	2.37	< .0001	2.50	< .0001		
**SBP**	0.0016	0.07			0.0018	0.01			-0.0027	< .0001	-0.0026	< .0001	0.0076	< .0001	0.0011	0.0008
**DBP**	0.0048	0.004	0.0051	0.01	0.0014	< .0001	0.0045	0.002	-0.0031	< .0001	0.0020	0.003	0.015	< .0001	0.0013	0.03
**Log FBG**	0.68	< .0001	0.58	0.004	0.58	< .0001			-0.077	0.13	0.65	< .0001	0.039	< .0001		
**Log PBG**	0.41	< .0001			0.50	< .0001			-0.17	< .0001	-0.16	0.0007	0.30	< .0001	0.22	< .0001
**HbA1c**	0.028	0.19			0.078	< .0001	0.072	< .0001	-0.03	< .0001	-0.046	< .0001	0.036	< .0001		

### Confounder-adjusted odds ratios for having adverse lipid concentrations by increment of different anthropometric parameters

Multiple logistic regressions were performed to determine which index (BMI, WC, WHpR or WHtR) was independently associated with high TC, high LDL-C, low HDL-C, hypercholesterolemia, hypertriglyceridemia and dyslipidemia. After adjustment for traditional risk factors of adverse lipid concentrations (such as sex, age, smoking, blood pressure, blood glucose), history of cardiovascular disease, lipid-lowering therapy and other anthropometric parameters, only BMI (per quartile increment) increased risks for prevalent high LDL-C (OR = 1.24; 95% CI = 1.08–1.42), low HDL-C (OR = 1.40; 95% CI = 1.26–1.57), hypercholesterolemia (OR = 1.39; 95% CI = 1.25–1.54), hypertriglyceridemia (OR = 1.20; 95% CI = 1.05–1.37), and dyslipidemia (OR = 1.36; 95% CI = 1.23–1.51) (Table 3).

**Table 3:** Confounder-adjusted odds ratios for having adverse lipid concentrations per quartile increment of different anthropometric parameters.

	BMI^*^	WC^#^	WHpR^&^	WHtR^△^
**High TC**				
**OR (95%CI)**	1.03 (0.89–1.27)	1.09 (0.86–1.38)	1.02 (0.86–1.20)	1.15 (0.93–1.43)
**P*-*value**	0.77	0.48	0.82	0.19
**High LDL-C**				
**OR (95%CI)**	**1.24 (1.08–1.42)**	0.99 (0.84–1.16)	0.97 (0.87–1.09)	1.05 (0.91–1.21)
**P*-*value**	**0.002**	0.87	0.60	0.47
**Low HDL-C**				
**OR (95%CI)**	**1.40 (1.26–1.57)**	1.05 (0.92–1.20)	1.03 (0.94–1.13)	0.96 (0.85–1.08)
**P*-*value**	**< .0001**	0.45	0.56	0.46
**Hypercholesterolemia**				
**OR (95%CI)**	**1.39 (1.25–1.54)**	1.09 (0.97–1.22)	1.06 (0.97–1.15)	1.07 (0.96–1.19)
**P*-*value**	**< .0001**	0.17	0.21	0.20
**Hypertriglyceridemia**				
**OR (95%CI)**	**1.20 (1.05–1.37)**	1.12 (0.95–1.31)	1.11 (0.99–1.24)	1.10 (0.95–1.26)
**P*-*value**	**0.008**	0.18	0.06	0.21
**Dyslipidemia**				
**OR (95%CI)**	**1.36 (1.23–1.51)**	1.08 (0.96–1.21)	1.07 (0.98–1.16)	1.08 (0.98–1.20)
**P*-*value**	**< .0001**	0.20	0.12	0.13

^*^Adjusted for sex, age, smoking, WC, HC, SBP, DBP, FBG, PBG, HbA1c, history of cardiovascular disease and lipid-lowering therapy

^#^Adjusted for sex, age, smoking, BMI, HC, SBP, DBP, FBG, PBG, HbA1c, history of cardiovascular disease and lipid-lowering therapy

^&^Adjusted for sex, age, smoking, BMI, SBP, DBP, FBG, PBG, HbA1c, history of cardiovascular disease and lipid-lowering therapy

^△^Adjusted for sex, age, smoking, BMI, HC, SBP, DBP, FBG, PBG, HbA1c, history of cardiovascular disease and lipid-lowering therapy.

Besides, we further explored associations between BMI and adverse lipid concentrations by stratifying individuals according to sex, age (<60 yr and ≥60 yr) and prevalence of MS. Whether in men or women, in younger or older people, in subjects without or with MS, per SD increment of BMI was independent risk factor for having low HDL-C, hypercholesterolemia and dyslipidemia. Among female or subjects with age ≥60 yr, increased BMI was also independently associated with having hypertriglyceridemia. In subjects without MS, increased BMI was associated with having high LDL-C. However, no any significant associations were found between increased BMI and prevalent high TC (Table 4).

**Table 4:** Odds ratios for having adverse lipid concentrations per SD increment of BMI in individuals stratified by sex, age (<60 yr and ≥60 yr) and prevalence of MS.

	Sex^*^		Age^#^		MS^&^	
	Male	Female	<60 yr	≥60 yr	Without	With
**SD of BMI (kg/m**^**2**^**)**	2.52	2.50	2.45	2.55	2.47	2.05
**High TC**						
**OR (95%CI)**	0.93 (0.56–1.58)	0.95 (0.72–1.26)	0.95 (0.66–1.36)	0.87 (0.62–1.24)	0.97 (0.72–1.31)	0.93 (0.65–1.33)
**P*-*value**	0.78	0.74	0.76	0.44	0.86	0.66
**High LDL-C**						
**OR (95%CI)**	1.31 (0.98–1.77)	1.22 (1.00–1.50)	1.16 (0.91–1.50)	1.22 (0.97–1.53)	**1.26 (1.03–1.55)**	1.13 (0.90–1.44)
**P*-*value**	0.07	0.06	0.24	0.09	**0.02**	0.29
**Low HDL-C**						
**OR (95%CI)**	**1.60 (1.32–1.96)**	**1.52 (1.26–1.85)**	**1.45 (1.18–1.80)**	**1.66 (1.39–1.99)**	**1.57 (1.29–1.91)**	**1.23 (1.03–1.46)**
**P*-*value**	**< .0001**	**< .0001**	**0.0006**	**< .0001**	**< .0001**	**0.02**
**Hypercholesterolemia**						
**OR (95%CI)**	**1.53 (1.25–1.88)**	**1.46 (1.25–1.72)**	**1.35 (1.12–1.63)**	**1.57 (1.33–1.87)**	**1.40 (1.20–1.64)**	**1.25 (1.04–1.50)**
**P*-*value**	**< .0001**	**< .0001**	**0.0016**	**< .0001**	**< .0001**	**0.02**
**Hypertriglyceridemia**						
**OR (95%CI)**	1.06 (0.81–1.39)	**1.31 (1.06–1.63)**	1.20 (0.95–1.52)	**1.30 (1.03–1.65)**	0.94 (0.71–1.24)	1.14 (0.94–1.37)
**P*-*value**	0.68	**0.01**	0.13	**0.03**	0.65	0.18
**Dyslipidemia**						
**OR (95%CI)**	**1.47 (1.20–1.80)**	**1.43 (1.23–1.67)**	**1.36 (1.14–1.63)**	**1.51 (1.28–1.79)**	**1.33 (1.15–1.55)**	**1.27 (1.05–1.54)**
**P*-*value**	**0.0002**	**< .0001**	**0.0007**	**< .0001**	**0.0002**	**0.02**

^*^Adjusted for age, smoking, WC, HC, SBP, DBP, FBG, PBG, HbA1c, history of cardiovascular disease and lipid-lowering therapy

^#^Adjusted for sex, age, smoking, WC, HC, SBP, DBP, FBG, PBG, HbA1c, history of cardiovascular disease and lipid-lowering therapy

^&^Adjusted for sex, age, smoking, WC, HC, SBP, DBP, FBG, PBG, HbA1c, history of cardiovascular disease and lipid-lowering therapy.

## Discussion

The prevalence of kinds of dyslipidemia was rapidly increasing during less than 10 years in China [3, 11]. It is well-known that weight reduction will enhance LDL-C lowering and reduce all of the risk factors of the metabolic syndrome. Overweight and obesity are also risk factors for elevated triglycerides and reduced HDL-C levels in the general population [4]. BMI is a measure of overall adiposity and defines the degree of obesity, whereas WC is a marker of central obesity. Several studies agreed that simple anthropometric measurements might do even better than the direct fat measures, such as computed tomography and bioelectrical impedance method, in the prediction of metabolic abnormalities and/or cardiovascular risk factors [12–15]. More attentions should be pay on these conventional and valuable anthropometric measurements. Moreover, latest survey considered that cardiometabolic disease (CMD) was also common in normal-weight persons and showed about 26.6%of the total Chinese population were of normal weight, but had mild-to-moderate CMD (245.2 million adults); 19.6% were of normal weight, but had severe CMD (180.5 million adults) [16]. The hung numbers were incredible. Our study tried to find the best anthropometric index to identify various lipid abnormalities in non-obese Chinese individuals aged more than 40 years.

Currently, it is widely accepted that visceral obesity is associated with an increased risk for some metabolic and cardiovascular diseases. Numerous studies suggested that WC or WHtR might be the better choice to identify metabolic risks as indices for body fat distribution [5, 12, 17]. It was considered that BMI could not distinguish fat from muscle mass [5]. Measures for central obesity were more sensitive to diet and training than BMI because increase of muscle mass might lead to little change of BMI but clear changes in WC and WHtR [18].

However, several different outcomes suggested that BMI was the better predictive index of prevalent diabetes, hypertension, and cardiovascular risk [19–22]. Besides, some studies showed the association between BMI and metabolic abnormality in Asian population recently. Hou XH *et al*. involved 46,024 Chinese participants aged more than 20 years and showed that BMI was associated with a higher risk of having hypertension or having hypertension plus dyslipidemia than WC [23]. Similar observations were made in 1891 subjects aged 21–74 years (Chinese 59.1%, Malay 22.2% and Indian 18.7%) in Singapore [24]. This was understood to be due to the BMI better reflecting body volume and mass, which were associated with blood viscosity and blood volume, and hence more closely related to blood pressure [23]. Another study confirmed that BMI, instead of WC or WHpR, produced a better role in predicting prevalent dyslipidemia in Chinese school-aged children with obesity by using stepwise disciminant analysis [25]. The varied results implied that each index might produce diverse effects on identifying dyslipidemia among different study populations.

The present study attempted to find the best one to discriminate prevalent dyslipidemia in a non-obese Chinese population aged more than 40 years. It seemed that BMI was the most correlated index with lipids than WC, WHpR and WHtR not only in stepwise regression analysis, but also in logistic regression analysis. It suggested that BMI, an index of overall adiposity, still played an important role on classifying risk of adverse lipid concentration in non-obese individuals. Besides, we found that BMI was most associated with low HDL-C and was less associated with high TC regardless of sex, age and prevalent MS. Some similar results showed the closely relationship between increased BMI and reduced HDL-C [26–27].

There are some limitations of the present study. First, the study was a cross-sectional study and the involved subjects were relatively limited. The interpretation of the results requires considerable caution. The effectiveness of BMI and other anthropometric measurements on predicting lipid or other metabolic abnormalities needs further validation in prospective studies done in different population. Second, the numbers of male participants were half of female in our study. There was potential gender bias. Third, the study described some characteristics in a population but lack of further researches on mechanism.

In conclusion, we found a significantly association between increasing BMI and prevalent dyslipidemia in non-obese Chinese population aged more than 40 years. As simple and non-invasive methods for a detection of metabolic abnormalities, anthropometric measurements could be efficiently used in clinical and epidemiologic fields. Due to the different meanings, both BMI and WC should be measured and monitored for metabolic risk assessment.

## Supporting information

S1 Table STROBE statement. Checklist of items that should be included in reports of observational studies.
(DOCX)
