# Supplementary material for: Associations between anthropometric parameters and lipid profiles in Chinese individuals with age ≥40 years and BMI <28kg/m2
Source: PLoS One. 2017 Jun 20;12(6):e0178343. doi: 10.1371/journal.pone.0178343 (PMC5478121; doi:10.1371/journal.pone.0178343)
Supplement: S1 Table — Checklist of items that should be included in reports of observational studies. (DOCX) [file pone.0178343.s001.docx]

**S1 Table. STROBE Statement.** Checklist of items that should be included in reports of observational studies

|  | Item No. | Recommendation | Page  No. | Relevant text from manuscript |
| --- | --- | --- | --- | --- |
| **Title and abstract** | 1 | (*a*) Indicate the study’s design with a commonly used term in the title or the abstract | 2 | Cross-sectional study |
|  |  | (*b*) Provide in the abstract an informative and balanced summary of what was done and what was found | 2 and 3 | Cross-sectional study of 4185 non-obese adults aged more than 40 years was conducted in Nanchang, Jiangxi province, China. In non-obese Chinese population aged more than 40 years, increasing BMI may better identify the prevalent dyslipidemia than other anthropometric measurements. |
| Introduction | | | |  |
| Background/rationale | 2 | Explain the scientific background and rationale for the investigation being reported | 4 | Elevated LDL-C, TG and reduced HDL-C levels are all associated with overweight and obesity. Usually, some simple anthropometric measurements are used to reflect excess body fat, such as body mass index (BMI), waist circumference (WC), waist-to-hip ratio (WHpR), and waist-to-height ratio (WHtR). BMI is mostly used to define obesity. However, abdominal obesity evaluated by WC or WHtR has got more public attention recently. |
| Objectives | 3 | State specific objectives, including any prespecified hypotheses | 4 and 5 | WC or WHtR are considered to be more correlated with metabolic risk factors than elevated BMI in several studies. However, a study among 1278 children did not find particular superiority for WC, especially for WHtR based on routine measurement of BMI in predicting metabolic or cardiovascular risk. It is still worth exploring the predictive effects by these anthropometric parameters in different population. In the present study, we compared the associations between various obesity indices and lipid profile in a non-obese (BMI <28kg/m^2^) Chinese population. |
| Methods | | | |  |
| Study design | 4 | Present key elements of study design early in the paper | 5 | The present cross-sectional study enrolled the subjects from communities and measured the obesity indexes and lipid concentrations. |
| Setting | 5 | Describe the setting, locations, and relevant dates, including periods of recruitment, exposure, follow-up, and data collection | 5 | During March to July 2011, the study was conducted in Ximazhuang and Guangrunmen communities form Xihu district of Nanchang, Jiangxi province, China. |
| Participants | 6 | (*a*) *Cohort study*—Give the eligibility criteria, and the sources and methods of selection of participants. Describe methods of follow-up  *Case-control study*—Give the eligibility criteria, and the sources and methods of case ascertainment and control selection. Give the rationale for the choice of cases and controls  *Cross-sectional study*—Give the eligibility criteria, and the sources and methods of selection of participants | 5 | In the recruiting phase, 5200 inhabitants aged more than 40 yr were invited by posters or telephones to participate in this program. From them, 4977 men and women attended to the study. Each subject had been registered with the identity card information. |
|  |  | (*b*)*Cohort study*—For matched studies, give matching criteria and number of exposed and unexposed  *Case-control study*—For matched studies, give matching criteria and the number of controls per case | - | N/A |
| Variables | 7 | Clearly define all outcomes, exposures, predictors, potential confounders, and effect modifiers. Give diagnostic criteria, if applicable | 7 | Based on NCEP ATP Ⅲ criteria , TC ≥ 6.22 mmol/L was defined as high TC，LDL-C ≥ 4.14 mmol/L was defined as high LDL-C, HDL-C < 1.04 was defined as low HDL-C. Hypercholesterolemia contains high TC and/or high LDL-C and/or low HDL-C. TG ≥ 2.26 mmol/L was defined as hypertriglyceridemia. Dyslipidemia was defined as hypercholesterolemia and/or hypertriglyceridemia and/or use of lipid lowering medications.  Metabolic syndrome (MS) was diagnosed as the presence of three or more of the following abnormal factors: 1. TG ≥ 1.7 mmol/L; 2. HDL-C < 1.0 mmol/L in men or < 1.3 mmol/L in women; 3. blood pressure ≥ 130/85 mmHg; 4. FBG ≥ 5.6 mmol/L or use of antidiabetic medications; 5. WC ≥ 85 cm in men or ≥ 80 cm in women. |
| Data sources/measurement | 8* | For each variable of interest, give sources of data and details of methods of assessment (measurement). Describe comparability of assessment methods if there is more than one group | 6 and 7 | A standard questionnaire was administered by trained staff to obtain information on demographic characteristics, medical history and lifestyle risk factors. Weight and height were determined in subjects wearing light clothing and no shoes. BMI was calculated as body weight in kilograms divided by body height squared in meters (kg/m^2^). WC was measured at the umbilical level in a standing position, whereas hip circumference (HC) was measured at the level of maximum extension of the buttocks. Twice sitting blood pressure measurements taken consecutively with 1-minute intervals using an automated electronic device (Omron Company, Dalian, China) were averaged for analysis.  All participants were under-taken a 75-g oral glucose tolerance test (OGTT) performed by a nurse. Blood samples were collected at 0 and 2 hours respectively by 2 specialized nurses. Serum TC, LDL-C, HDL-C and TG were measured using chemiluminescence methods, fasting blood glucose (FBG) and postprandial blood glucose (PBG) were measured using the glucose oxidase method on the same autoanalyser (Roche, Basel, Switzerland). The HbA1c level was measured by high-performance liquid chromatography (BIO-RAD Company, USA). |
| Bias | 9 | Describe any efforts to address potential sources of bias | 5 | We tried our best to obtain sufficient informed consent from inhabitants and avoid the age or gender bias as far as possible. |
| Study size | 10 | Explain how the study size was arrived at | 5 and 6 | The deleted individuals included incomplete questionnaire (3 of them missing gender, 16 missing age, 2 missing height and 2 missing weight), undesirable age (79 of them aged less than 40 yr) and extremum (73 with incredible anthropometric parameters and 11 with wrong laboratory values). 606 obese participants (BMI ≥28kg/m^2^) were excluded from this study and at last, 4185 subjects were included in the final analysis. |

Continued on next page

| Quantitative variables | 11 | Explain how quantitative variables were handled in the analyses. If applicable, describe which groupings were chosen and why | 7 | Continuous variables were presented as means ± standard deviations (SD) or medians (interquartile ranges). FBG, PBG and TG levels were logarithmically transformed to achieve a normal distribution. All categorical variables were presented as numbers (proportions). The subjects were divided to different groups by sex. |
| --- | --- | --- | --- | --- |
| Statistical methods | 12 | (*a*) Describe all statistical methods, including those used to control for confounding | 7 and 8 | Comparisons of means and proportions were performed with Student’s *t*-test and Chi-squared test. Stepwise regression analysis was used to identify the association between anthropometric parameters and lipid profiles. The confounder-adjusted odds ratios (ORs) and 95% confidence intervals (CIs) were examined by logistic regression analyses. A *p* value of less than 0.05 was considered to be statistically significant. |
|  |  | (*b*) Describe any methods used to examine subgroups and interactions | 7 | Furthermore, we explored some subgroups by stratifying individuals according to sex, age (< 60yr and ≥ 60yr) and prevalence of MS. |
|  |  | (*c*) Explain how missing data were addressed | - | N/A |
|  |  | (*d*) *Cohort study*—If applicable, explain how loss to follow-up was addressed  *Case-control study*—If applicable, explain how matching of cases and controls was addressed  *Cross-sectional study*—If applicable, describe analytical methods taking account of sampling strategy | 7 | Cluster sampling method was used for the investigation. |
|  |  | (*e*) Describe any sensitivity analyses | - | N/A |
| Results | | | | |
| Participants | 13* | (a) Report numbers of individuals at each stage of study—eg numbers potentially eligible, examined for eligibility, confirmed eligible, included in the study, completing follow-up, and analysed | 8 | The present data included 4185 participants (33.52% men and mean age 61 ± 10 years). |
|  |  | (b) Give reasons for non-participation at each stage | - | N/A |
|  |  | (c) Consider use of a flow diagram | 8 | The flow diagram is showed in Fig 1. |
| Descriptive data | 14* | (a) Give characteristics of study participants (eg demographic, clinical, social) and information on exposures and potential confounders | 8 | The present data included 4185 participants (33.52% men and mean age 61 ± 10 years). The overall prevalence of high TC, high LDL-C, low HDL-C, hypercholesterolemia, hypertriglyceridemia and dyslipidemia were 15.68%, 27.98%, 20.12%, 44.01%, 21.98% and 49.06% respectively. 423 subjects (10.11%) had lipid-lowering therapy. Compared with women, men had a signiﬁcantly higher BMI, WC, WHpR, SBP, DBP, FBG, PBG, HbA1c and TG concentration, but smaller WHtR, lower TC, LDL-C and HDL-C concentrations. Besides, more men had the habit of smoking and had dyslipidemia (all *p* value < 0.05). However, the comparison for having MS was not statistically different between both genders (*p* value = 0.45) (Table 1). |
|  |  | (b) Indicate number of participants with missing data for each variable of interest | - | N/A |
|  |  | (c) *Cohort study*—Summarise follow-up time (eg, average and total amount) | - | N/A |
| Outcome data | 15* | *Cohort study*—Report numbers of outcome events or summary measures over time | - | N/A |
|  |  | *Case-control study—*Report numbers in each exposure category, or summary measures of exposure | - | N/A |
|  |  | Cross-sectional study—Report numbers of outcome events or summary measures | 9 | Linear regression analysis revealed that sex, smoking status, BMI, WHtR, DBP and FBG were significantly related to TC, LDL-C, HDL-C and TG concentration. WC, WHpR, SBP and HbA1c were only significantly related with LDL-C, HDL-C and TG concentration (Table 2). Multiple stepwise regression analysis revealed that apart from traditional risk factors of adverse lipid concentrations, BMI, WC, WHpR and WHtR remained independent relationships with HDL-C. Besides, BMI was also an independent determinant of LDL-C and TG, WC and WHtR were also independent determinants of TC, WHpR was independent related with TG (Table 2). |
| Main results | 16 | (*a*) Give unadjusted estimates and, if applicable, confounder-adjusted estimates and their precision (eg, 95% confidence interval). Make clear which confounders were adjusted for and why they were included | 9 and 10 | Multiple logistic regressions were performed to determine which index (BMI, WC, WHpR or WHtR) was independently associated with high TC, high LDL-C, low HDL-C, hypercholesterolemia, hypertriglyceridemia and dyslipidemia. After adjustment for traditional risk factors of adverse lipid concentrations (such as sex, age, smoking, blood pressure, blood glucose), history of cardiovascular disease, lipid-lowering therapy and other anthropometric parameters, only BMI (per quartile increment) increased risks for prevalent high LDL-C (OR = 1.24; 95% CI = 1.08-1.42), low HDL-C (OR = 1.40; 95% CI = 1.26-1.57), hypercholesterolemia (OR = 1.39; 95% CI = 1.25-1.54), hypertriglyceridemia (OR = 1.20; 95% CI = 1.05-1.37), and dyslipidemia (OR = 1.36; 95% CI = 1.23-1.51) (Table 3). |
|  |  | (*b*) Report category boundaries when continuous variables were categorized | 8 | The category boundaries of continuous variables are showed in Table 1. |
|  |  | (*c*) If relevant, consider translating estimates of relative risk into absolute risk for a meaningful time period | - | N/A |

Continued on next page

| Other analyses | 17 | Report other analyses done—eg analyses of subgroups and interactions, and sensitivity analyses | 10 | Besides, we further explored associations between BMI and adverse lipid concentrations by stratifying individuals according to sex, age (< 60yr and ≥ 60yr) and prevalence of MS. Whether in men or women, in younger or older people, in subjects without or with MS, per SD increment of BMI was independent risk factor for having low HDL-C, hypercholesterolemia and dyslipidemia. Among female and subjects with age ≥ 60yr, increased BMI was also independently associated with having hypertriglyceridemia. In subjects without MS, increased BMI was associated with having high LDL-C. However, no any significant associations were found between increased BMI and prevalent high TC (Table 4). |
| --- | --- | --- | --- | --- |
| Discussion | | | | |
| Key results | 18 | Summarise key results with reference to study objectives | 12 | The present study attempted to find the best one to discriminate prevalent dyslipidemia in a non-obese Chinese population aged more than 40 years. It seemed that BMI was the most correlated index with lipids than WC, WHpR and WHtR not only in stepwise regression analyses, but also in logistic regression analyses. It suggested that BMI, an index of overall adiposity, still played an important role on classifying risk of adverse lipid concentration in non-obese individuals. Besides, we found that BMI was most associated with low HDL-C and was less associated with high TC regardless of sex, age and prevalent MS. |
| Limitations | 19 | Discuss limitations of the study, taking into account sources of potential bias or imprecision. Discuss both direction and magnitude of any potential bias | 12and 13 | There are some limitations of the present study. First, the study was a cross-sectional study and the involved subjects were relatively limited. The interpretation of the results requires considerable caution. The effectiveness of BMI and other anthropometric measurements on predicting lipid or other metabolic abnormalities needs further validation in prospective studies done in different population. Second, the numbers of male participants were half of female in our study. There was potential gender bias. Third, the study described some characteristics in a population but lack of further researches on mechanism. |
| Interpretation | 20 | Give a cautious overall interpretation of results considering objectives, limitations, multiplicity of analyses, results from similar studies, and other relevant evidence | 11 and 12 | However, several different outcomes suggested that BMI was the better predictive index of prevalent diabetes, hypertension, and cardiovascular risk. Besides, some studies showed the association between BMI and metabolic abnormality in Asian population recently. Hou XH *et al.* involved 46,024 Chinese participants aged more than 20 years and showed that BMI was associated with a higher risk of having hypertension or having hypertension plus dyslipidemia than WC. Similar observations were made in 1891 subjects aged 21-74 years (Chinese 59.1%, Malay 22.2% and Indian 18.7%) in Singapore. This was understood to be due to the BMI better reflecting body volume and mass, which were associated with blood viscosity and blood volume, and hence more closely related to blood pressure. Another study confirmed that BMI, instead of WC or WHpR, produced a better role in predicting prevalent dyslipidemia in Chinese school-aged children with obesity by using stepwise disciminant analysis. The varied results implied that each index might produce diverse effects on identifying dyslipidemia among different study populations. |
| Generalisability | 21 | Discuss the generalisability (external validity) of the study results | 13 | As simple and non-invasive methods for a detection of metabolic abnormalities, anthropometric measurements could be efficiently used in clinical and epidemiologic fields. Due to the different meanings, both BMI and WC should be measured and monitored for metabolic risk assessment. |
| Other information | |  | | |
| Funding | 22 | Give the source of funding and the role of the funders for the present study and, if applicable, for the original study on which the present article is based |  | This study was supported by the grants from Chinese Center for Disease Control and Prevention (Number 2009-103, Recipient: Jia Weiping), Health and Family Planning Commission of Jiangxi Province (Number 2010-26, Recipient: Tu ping), Jiangxi Medical Association (Number 2013-8, Recipient: Tu ping), Jiangxi Committee of Science and Technology (Number 20133ACI90016, Recipient: Tu ping), Health and Family Planning Commission of Nanchang City, Jiangxi Province (Number 2010-27，2010-38, 2011-6 and 2011-33, Recipient: Tu ping). |

*Give information separately for cases and controls in case-control studies and, if applicable, for exposed and unexposed groups in cohort and cross-sectional studies.

**Note:** An Explanation and Elaboration article discusses each checklist item and gives methodological background and published examples of transparent reporting. The STROBE checklist is best used in conjunction with this article (freely available on the Web sites of PLoS Medicine at http://www.plosmedicine.org/, Annals of Internal Medicine at http://www.annals.org/, and Epidemiology at http://www.epidem.com/). Information on the STROBE Initiative is available at www.strobe-statement.org.
